# Hepatocellular carcinoma: a clinicopathological study of 64 cases

**DOI:** 10.11604/pamj.2017.27.41.9584

**Published:** 2017-05-16

**Authors:** Faten Limaiem, Marwa Bouhamed, Ghada Sahraoui, Sabeh Mzabi

**Affiliations:** 1 Université de Tunis El Manar, Faculté de Médecine de Tunis, 1007

**Keywords:** Liver, hepatocellular carcinoma, cancer, pathology

## Abstract

Hepatocellular carcinoma (HCC) is the most common of all liver cancers and is a major worldwide public health problem. The aim of this study was to provide an updated overview on clinicopathological features, treatment and outcome of HCC. In our retrospective study, we reviewed 64 cases of HCC that were diagnosed at the pathology department of Mongi Slim hospital over a fifteen-year period (2000- 2014). Relevant clinical information and microscopic slides were retrospectively reviewed. Our study group included 38 men and 26 women (sex ratio M/F = 1,26) aged between 8 and 83 years (mean = 56,64 years). The presenting clinical symptoms were dominated by abdominal pain (n=34), followed by altered general health (n=25) and jaundice (n=4). Fifty-five patients underwent surgical treatment. Liver transplantation was performed in two cases and transarterial chemoembolization was achieved in seven cases. Histopathological examination of the surgical or biopsy specimen established the diagnosis of conventional HCC in 55 cases, fibrolamellar carcinoma in 6 cases and clear cell HCC in 3 cases. Seven patients with HCC died postoperatively. Local recurrence of the tumour occurred in three cases and two patients had distant metastases postoperatively. The other patients are still being followed-up. Hepatocellular carcinoma is associated with a high rate of mortality because of early invasion, widespread metastasis and lack of effective therapeutic modalities. Accurate diagnosis and staging of these tumours is critical for optimal treatment planning and for determining prognosis.

## Introduction

Hepatocellular carcinoma (HCC) is the fifth most common cancer and the third cause of cancer-related deaths in the world. It is the most common cause of death in patients with cirrhosis [1, 2]. In this paper, we report our experience with HCC over the past 15 years. Our aim was to analyze epidemiological characteristics, clinical symptoms, radiological features, treatment and outcomes of 64 patients who were surgically treated at our institution. Our results are analyzed in comparison to a review of the literature.

## Methods

We undertook a retrospective study of 64 patients who were operated on for HCC at the General Surgery Department of Mongi Slim hospital of Tunis between February 2000 and November 2014. The cases were retrieved from the files of the registry of surgery of the same hospital. Medical records were scrutinized for epidemiological characteristics, predisposing factors, initial manifestations of the disease, methods of diagnosis, laboratory findings, surgical or palliative therapy and overall morbidity and mortality. Diagnosis of HCC was based upon clinical, imaging and histopathological findings. All patients underwent imaging evaluation during the preoperative period. All specimens were surgically obtained. Tissues were fixed in 10% phosphate buffered formaldehyde, embedded in paraffin and sections were prepared for routine light microscopy after staining with haematoxylin and eosin. For clear cell HCC, immunohistochemical analysis was performed using the avidin-biotin complex technique with antibodies against Hepatocyte Specific Antigen (HepPar1). Patient confidentiality was maintained.

## Results

**Clinical findings**: Our study group included 38 male and 26 female patients (sex-ratio M/F = 1,26) between 8 and 83 years of age (mean = 56,64 years). Fibrolamellar carcinomas comprised two male and four female patients (sex-ratio M/F = 0,5) aged between 20 and 38 years (mean = 33,16 years). The presenting clinical symptoms were dominated by abdominal pain (n=34), followed by altered general health (n=25), jaundice (n=4) and fever (n=1).

**Biological tests**: Preoperative serum alpha-fetoprotein levels were performed in 50 cases. They were elevated in 40 cases and within normal range in 10 cases. Preoperative serum carbohydrate antigen CA 19-9 levels were performed in eight cases. They were elevated in four cases (> 100 U/ml) and within normal range in four cases. Preoperative serum CEA levels were performed in fourteen cases. They were slightly elevated (> 5ng /ml) in three cases and within normal range in four cases. Hepatitis B and C serology were performed in 50 cases. Hepatitis C serology was positive in 15 cases and hepatitis B serology was positive in 14 cases.

**Radiological findings and localization of hepatocellular carcinoma**: Diagnostic imaging techniques included ultrasonography in all cases, CT scan in 53 cases and abdominal MRI in 24 cases. Based on CT scan findings, preoperative diagnosis of HCC was accurately made in 50 cases (94, 33%).

**Treatment**: Fifty-five patients underwent surgical treatment. Liver transplantation was performed in two cases and transarterial chemoembolization was achieved in seven cases.

**Figure 1:**
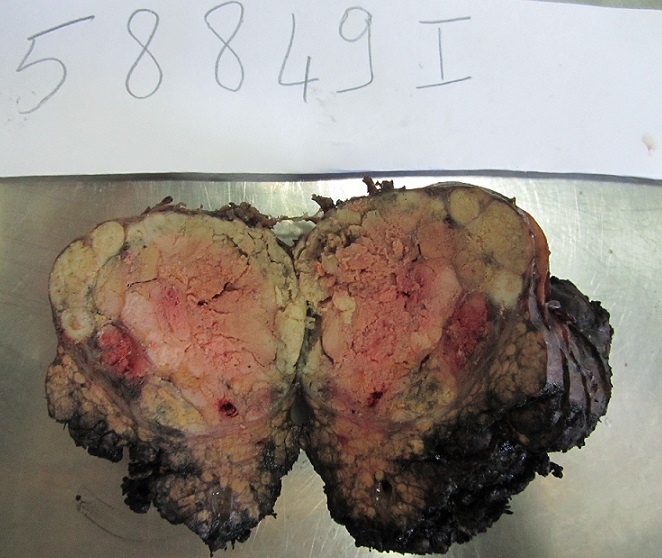
Macroscopic findings of hepatocellular carcinoma; a well delineated yellowish and encapsulated nodule arising in a cirrhotic liver

**Figure 2:**
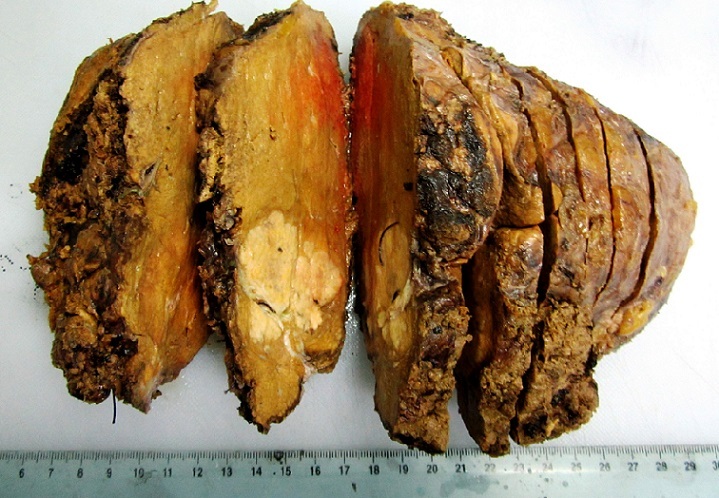
Macroscopic findings of hepatocellular carcinoma; an ill-defined tumour arising in a non-cirrhotic liver

**Figure 3:**
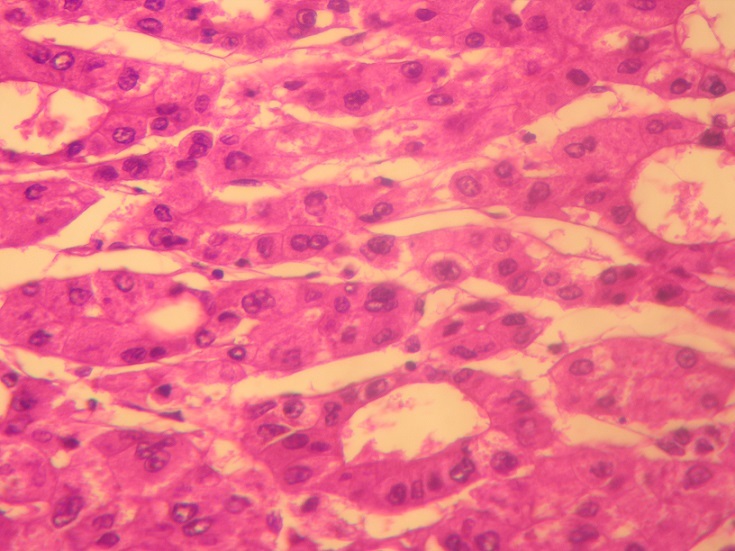
Moderately differentiated hepatocellular carcinoma; trabecular and pseudoglandular pattern (Hematoxylin and eosin, magnification × 400)

**Figure 4:**
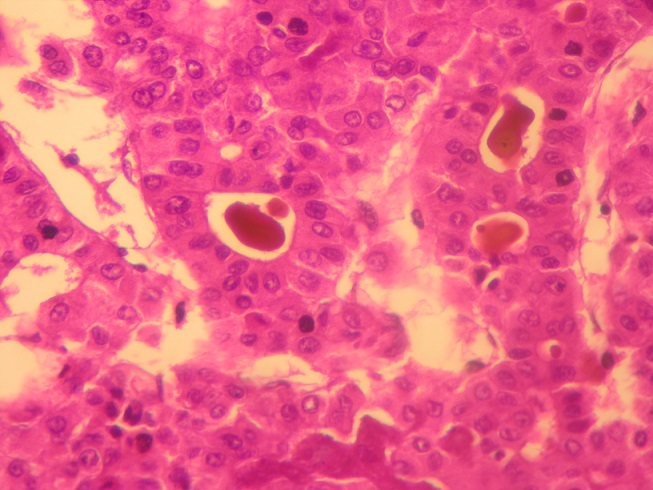
Moderately differentiated hepatocellular carcinoma; pseudoglandular pattern with bile plugs (Hematoxylin and eosin, magnification × 400)

**Pathologic Findings**: In our series, HCC ranged in size from 0,5 to 17 cm (mean = 5,75 cm). The tumour was multifocal in 10 cases. Fourty-nine cases of HCC developed in a cirrhotic liver (76,56%) Figure 1. The six cases of fibrolamellar carcinoma arose in a non-cirrhotic liver. On cut section, the tumours were well delineated in 40 cases and with ill-defined borders in 24 cases Figure 2. They were firm, gray-white to tan, yellowish or greenish. Histopathological examination of the surgical or biopsy specimen established the diagnosis of conventional HCC in 55 cases Figure 3, Figure 4, fibrolamellar carcinoma in 6 cases and clear cell HCC in 3 cases. Lymph node metastases were histologically detected in two cases whereas vascular invasion was noted in seven cases. Immunohistochemical study was performed in three cases of clear cell HCC so as to confirm diagnosis and to rule out metastasis. In the three cases, the tumour cells were positive for HepPar1.

**Operative morbidity and postoperative complications**: Seven patients with HCC died postoperatively. Other postoperative complications included hemorrhagic shock (n=6), peritonitis (n=2), renal failure (n =1) and sepsis (n=2).

**Follow-up and evolution**: The mean follow-up period of our patients was 26 months. Eight patients were lost to follow-up. Four patients died after a mean follow-up period of 10 months. Local recurrence of the tumour occurred in three cases and two patients had distant metastases postoperatively. The other patients are still being followed-up.

## Discussion

The incidence of HCC varies in different countries, depending on the prevalence of major causes, namely chronic viral hepatitis (hepatitis B and C) or other chronic liver diseases (fatty liver disease, alcohol, hemochromatosis, and α-1-antitrypsin deficiency). Hepatocellular carcinoma occurs more frequently in older than in young individuals and affects men more often than women at a ratio of 2:1 to 4:1 [3]. In our series, mean age at presentation was 56 years with a male predominance (sex-ratio M/F = 1,26). The presenting signs and symptoms in patients with HCC may be caused by the tumour itself or by the advanced stage of chronic liver disease predisposing to malignancy. Thus symptoms include abdominal pain, general malaise, anorexia or weight loss and nausea or vomiting [4]. Common clinical signs include hepatomegaly, ascites, fever, jaundice and splenomegaly. In our series, the presenting clinical symptoms were dominated by abdominal pain (n=34), followed by altered general health (n=25), jaundice (n=4) and fever (n=1). The laboratory findings are partly determined by the underlying liver disease and may be reflected in changes in the results of blood tests for liver enzymes, which are not, however, HCC-specific. A significantly raised serum level of alpha-fetoprotein of > 400ng/ml, or a continuous rise even if < 100ng/ml, strongly suggests HCC [5]. Imaging studies are important in patient management for the identification and localization of HCC. Useful techniques include ultrasonography (US), colour Doppler US, contrast-enhanced US, computed tomography (CT), lipiodol CT, magnetic resonance imaging (MRI), angiography, CT during hepatic arteriography and CT during arterial portography. The standard imaging techniques are US, CT and MRI. In most cases, these allow detection and staging of HCC. Typical findings associated with HCC that have progressed are arterial hypervascularity with venous wash-out on dynamic CT, dynamic MRI or contrast-enhanced US [6]. Treatment of HCC is multidisciplinary. The involvement of hepatologists, oncologists, radiologists as well as surgeons, is necessary to provide the most up-to-date care and to ensure the best outcomes. Surgical resection and orthotopic liver transplantation are the only potentially curative treatments for HCC. However, the coexistence of chronic liver diseases and the insidious nature of HCC make it unresectable in most patients. In these cases, treatment normally consists of local ablation therapies using ethanol injection or radiofrequency irradiation as well as transarterial chemoembolization [7].

At gross examination, HCC may display a nodular, infiltrative, or diffuse macroscopic pattern. The nodular (expanding) pattern is the most common type. It is typically seen in association with cirrhosis. Tumor nodules may be solitary or multiple across the liver. The infiltrative (massive) pattern is usually characterized by a single large mass that occupies a substantial portion of the liver. The lesion is poorly circumscribed with ill-defined, invasive borders. The diffuse pattern is the least common and represents widespread infiltration by numerous small nodules that virtually replace the entire liver. Pedunculation is noted in rare instances, presumably reflecting origin from an accessory hepatic lobe [8]. Histologically, HCC consist of tumour cells that usually resemble hepatocytes. The stroma is composed of sinusoid-like blood spaces lined by a single layer of endothelial cells. Unlike the sinusoidal endothelial cells in normal liver tissue, those of HCC show changes of capillarization. HCC vary architecturally and cytologically. The different architectural patterns and cytological variants frequently occur in combination. Immunohistochemically, HCC is characterized by cytoplasmic positivity with antibodies to HepPar1. It has been reported that 90% of all HCC are positive for HepPar1. Canalicular patterns may be seen with immunohistochemical staining with polyclonal antibodies to carcinoembryonic antigen (CEA) or antibodies to CD10. HCC is also often positive for AFP, fibrinogen and keratins 8 and 18 but usually negative for keratins 19 and 20 and epithelial membrane antigen [9]. Fibrolamellar carcinomas are distinctive liver cancers of children and young adults that differ from classical HCC at the clinical, histological and molecular levels. They account for 0,5 - 9% of primary liver cancer in various case series and occur in young adults and have no association with cirrhosis or other known risk factors. Fibrolamellar carcinoma arises in non-cirrhotic livers as it was the case in our series. This variant tends to present as well circumscribed, nodular, yellow-to-brown tumours with extensive fibrosis grossly. Some tumours may present with a “central scar.” Histologically, this variant is characterized by the presence of dense bands of lamellar fibrous tissue separating the tumor cells that are typically polygonal and exhibit large nuclei with prominent nucle¬oli [10]. The prognosis of patients with HCC is still unsatisfactory. Even patients that are eligible for surgical resection exhibit a 5-year survival rate of only 50% [11]. Patients with more advanced disease exhibit a median survival time of only 6-9 months and this reduces to only a few months in untreated patients [12]. Spontaneous regression of HCC is exceedingly rare with only 47 cases reported in literature previously [13].

## Conclusion

In summary, this retrospective study from Tunisia provides an overview on clinical symptoms, radiological features, treatment and outcome in 64 patients with HCC. Hepatocellular carcinoma is associated with a high rate of mortality because of early invasion, widespread metastasis and lack of effective therapeutic modalities. Accurate diagnosis and staging of these tumours is critical for optimal treatment planning and for determining prognosis. With the increasing knowledge of the molecular pathogenesis of this disease, there is hope for nonsurgical alternatives in the future, especially targeted therapies.

### What is known about this topic

- Hepatocellular carcinoma is associated with a high rate of mortality because of early invasion, widespread metastasis and lack of effective therapeutic modalities;
- Accurate diagnosis and staging of hepatocellular carcinoma is critical for optimal treatment planning and for determining prognosis.

### What this study adds

- This retrospective study from Tunisia provides an overview on clinical symptoms, radiological features, treatment and outcome in 64 Tunisian patients with hepatocellular carcinoma;
- As reported in literature, prognosis of hepatocellular carcinoma is very poor in Tunisia since this tumour is detected at advanced stages. In developing countries like Tunisia, screening of this disease namely in patients with chronic hepatitis B or C may be helpful in detecting hepatocellular carcinoma at an earlier stage. With the increasing knowledge of the molecular pathogenesis of this disease, there is hope for nonsurgical alternatives in the future, especially targeted therapies.

## Competing interests

The author declare no competing interests.
